# Fatal arrhythmia associated with novel coronavirus 2019 infection: Case report and literature review

**DOI:** 10.1097/MD.0000000000037894

**Published:** 2024-04-19

**Authors:** Hongyun Shu, Qiaowen Li, Xiaoyong Zhang, Guojun Zhao, Yaqian Cui, Xiyan Zhu

**Affiliations:** a Department of Cardiovascular Medicine, Affiliated Qingyuan Hospital, Guangzhou Medical University, Qingyuan People’s Hospital, Guangdong, China; b Guangzhou Medical University, Guangzhou, China; c Institute of Gerontology, Guangzhou Geriatric Hospital, Guangzhou Medical University, Guangzhou, China.

**Keywords:** COVID-19, high-degree atrioventricular block, malignant arrhythmias

## Abstract

**Rationale:**

The novel coronavirus of 2019 (COVID-19) has inflicted significant harm on the cardiovascular system. Patients presenting with fatal chronic arrhythmias after severe acute respiratory syndrome coronavirus 2 (SARS-CoV-2) infection are rare, arrhythmia caused by SARS-CoV-2 infection need to be taken seriously.

**Patient concerns:**

Three female patients were admitted to the hospital with syncopal symptoms. Previously, they had been identified to have COVID-19 infection and none of the patients had a preexisting history of arrhythmia, and upon hospital admission, no electrolyte imbalances associated with arrhythmias were observed. However, following SARS-CoV-2 infection, patients exhibit varying degrees of syncope symptoms.

**Diagnoses:**

A high-degree atrioventricular block was diagnosed after a comprehensive evaluation of the patient’s clinical manifestations and electrocardiogram (ECG) performance.

**Interventions:**

We performed ECG monitoring of the patient and excluded other causes of arrhythmia. The patient was discharged from the hospital after permanent pacemaker implantation and symptomatic treatment.

**Outcomes:**

The outpatient follow-ups did not reveal a recurrence of syncope or complications related to the pacemaker in any of the three patients.

**Lessons:**

Some patients did not exhibit any obvious respiratory symptoms or signs following SARS-CoV-2 infection. This suggests that the cardiac conduction system may be the preferred target for some SARS-CoV-2 variants. Therefore, in addition to investigating the causes of malignant arrhythmias, special attention should be paid to SARS-CoV-2 infection in patients with developing arrhythmias. Additionally, permanent pacemaker implantation may be the most suitable option for patients who already have malignant arrhythmias.

## 1. Introduction

Before the novel coronavirus of 2019 (COVID-19) pandemic, infections with preexisting coronaviruses such as severe acute respiratory syndrome (SARS) and middle east respiratory syndrome (MERS) were associated with various types of arrhythmias. A retrospective analysis of COVID-19 patients worldwide found that 18% of patients suffered from arrhythmias. The majority of these cases were atrial and supraventricular arrhythmias, while ventricular and bradyarrhythmias occurred less frequently.^[1]^ These findings are supported by three clinical cases that demonstrated the development of uncommon arrhythmias after SARS-CoV-2 infection. Interestingly, some patients did not exhibit obvious respiratory symptoms or signs after infection but presented with various clinical manifestations of arrhythmia as the initial symptom. Therefore, when diagnosing malignant arrhythmias, it is crucial to consider the possibility of novel coronavirus infection.

## 2. Case description

### 2.1. Case 1

**Figure 1.**
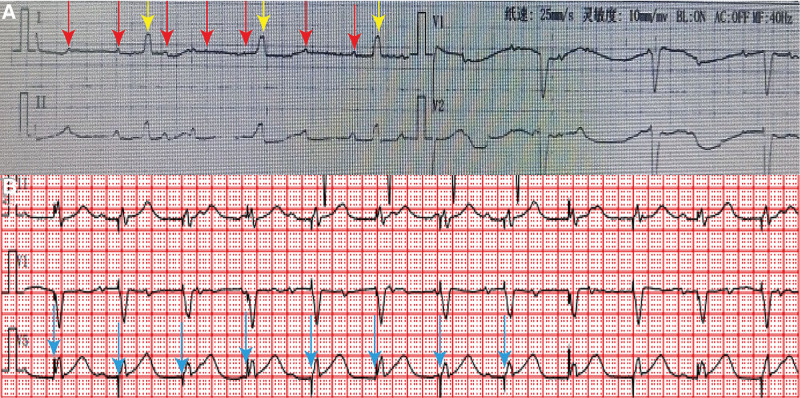
(A) ECG at presentation: AV block, PR interval of 120 ms, and heart rate of 40 bpm. (B) ECG after pacemaker implantation: the cardiac electrical axis of the left deviation, artificial ventricular pacing electrocardiogram was VVI mode of operation, and pacemaking function did not show any abnormality. ECG = electrocardiogram.

An 88-year-old woman of Han ethnicity with syncope was admitted to our hospital’s emergency department. Her electrocardiogram (ECG) indicated sinus rhythm, third-degree atrioventricular (AV) block, a PR interval of 120 ms, and a heart rate of 40 bpm (Fig. 1A). As part of the routine examination, SARS-CoV-2 nucleic acid test results were positive. In the emergency room, her heart rate did not show any significant improvement after atropine treatment. Shortly thereafter, she experienced further syncope, accompanied by shortness of breath and loss of consciousness. Cardiopulmonary resuscitation was promptly performed, leading to the recovery of consciousness. Subsequently, she was transferred to the general ward for further treatment after the implantation of a temporary pacemaker. Upon being downgraded to the general ward, the results of additional tests showed cardiac troponin I levels of 0.048 µg/L (reference range: 0–0.014 µg/L), N-terminal pro-B-type natriuretic peptide levels greater than 35000.00 pg/mL(reference range: <1800 pg/mL), C-reactive protein levels of 14.64 mg/L (reference range: 0–10 mg/L), and procalcitonin levels of 1.76 ng/mL (reference range: 0–0.05 ng/mL). These laboratory test results suggest that the patient had severe cardiac and hepatic insufficiency, likely caused by inadequate organ perfusion after cardiac arrest. Chest computed tomography revealed inflammation in the lower lobes of the lungs and a small amount of bilateral pleural effusion in the chest cavity. Echocardiography indicated moderate mitral valve regurgitation, severe tricuspid regurgitation, abnormal segmental motion of the left ventricular wall, and normal left ventricular systolic function. After treatment with diuresis, liver protectants, and anti-heart failure medication, the patient’s general condition improved. Given the advanced age of the patient and high risk of recurrent cardiovascular accidents, a permanent pacemaker was implanted. Postoperative electrocardiography revealed an artificial ventricular pacing electrocardiogram (VVI) mode of pacemaker implantation (Fig. 1B). The patient had smooth postoperative recovery and was discharged.

### 2.2. Case 2

**Figure 2.**
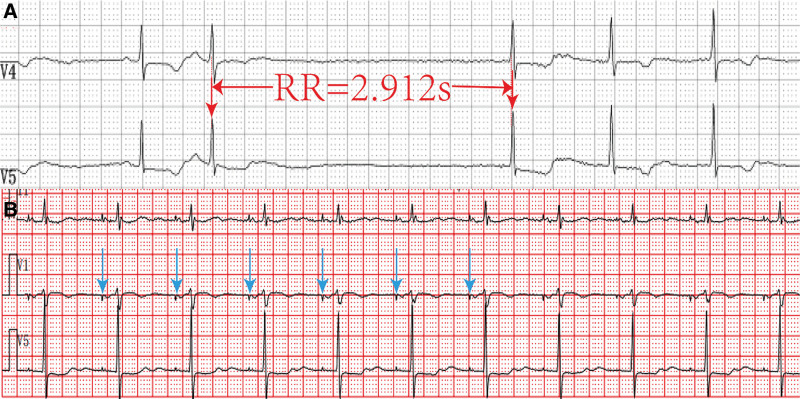
(A) Ambulatory electrocardiogram: sinus arrest (longest RR interval, 2.921 seconds) with the lowest ventricular rate of 27 bpm. (B) ECG after pacemaker implantation: AAI pacing mode with a pacing heart rate of 60 bpm. ECG = electrocardiogram.

A 76-year-old woman of Han ethnicity was admitted to our hospital because of sporadic syncopal episodes. She began experiencing dizziness 10 days prior, and her SARS-CoV-2 nucleic acid test results were positive. The patient had sporadic dizziness and a brief dark haze, but no apparent signs of respiratory infection. In our hospital, electrocardiogram indicated sinus arrest, atrial premature beats, and changes in the ST-T interval. The longest recorded RR interval was 1.8 seconds. D-dimer levels were 7.91 mg/L (reference range: 0–0.5 mg/L), and cardiac ultrasound confirmed normal left ventricular systolic function (68%). A 24-hour Holter ECG suggested sinus arrest (with the longest RR interval of 2.912 seconds) and sinus bradycardia (with the slowest heart rate of 27 beats/min) (Fig. 2A). Throughout her hospital stay, the patient experienced recurrent dizziness and discomfort, indicating the need for pacemaker implantation. Following pacemaker implantation, the patient no longer experienced syncope and was discharged from the hospital with improvements in all indices. Repeat ECG was unremarkable before discharge (Fig. 2B).

### 2.3. Case 3

**Figure 3.**
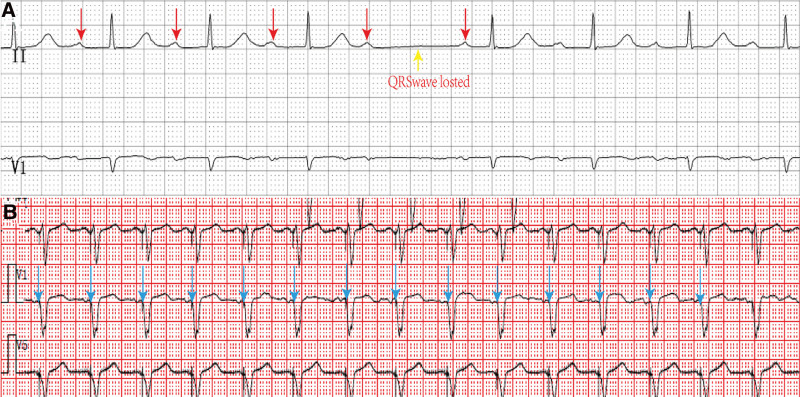
(A) ECG at presentation: second-degree type I atrioventricular block, 53 bpm. (B) ECG after pacemaker implantation: VAT pacing mode with a pacing heart rate of 88 bpm. ECG = electrocardiogram.

A 69-year-old Han woman with a history of nasopharyngeal cancer surgery and no history of hypertension, diabetes mellitus, or cardiac disease was admitted to the hospital with dizziness and weakness for half a month and fainting for one day. Her electrocardiogram (ECG) showed a second-degree atrioventricular block (Fig. 3A). The patient had a mild cough and sputum expectoration half a month ago, and the COVID-19 nucleic acid detection results were positive without other special manifestations. After admission, the initial laboratory test results were normal, including the cardiac enzyme spectrum, coagulation function, and liver and kidney function. The patient remained symptomatic during the hospitalization. After the implantation of a permanent pacemaker (Fig. 3B), the patient had no recurrence of dizziness or syncope, and no complications related to pacemaker implantation or other symptoms occurred in the outpatient clinic.

## 3. Discussion

In this case series, we describe three patients who were admitted to the hospital with syncopal symptoms and subsequent events of malignant arrhythmias. These patients had no prior history of cardiac arrhythmias or other cardiac diseases, and their post-hospitalization workups did not suggest any evidence of contributing factors, such as serious infections, hydroelectrolytic disorders, and thyroid disorders. However, it is worth noting that a study conducted at Zhongnan Hospital of Wuhan University found that 16.7% of 138 COVID-19-infected patients experienced different degrees of arrhythmia, with this percentage increasing to 44% in ICU patients.^[2]^ We found that patients with tachyarrhythmias, such as atrial fibrillation and sinus tachycardia, accounted for a higher proportion of patients with COVID-19, whereas bradyarrhythmias were less frequent.^[3]^ Another study indicated that patients with severe cardiac arrest after SARS-CoV-2 infection had higher blood potassium levels than non-severe patients at the time of admission.^[4]^ We do not believe that the malignant arrhythmias in the 3 patients were secondary to myocarditis, as none of them showed abnormalities in the markers of cardiac injury after admission, and case 1 showed normal cardiac troponin I in subsequent reviews. A significant finding from our case series was that most patients developed complete atrioventricular block after myocarditis due to SARS-CoV-2 infection.^[5]^ These findings suggest that SARS-CoV-2 infection may be the direct cause of arrhythmias in these three patients. Furthermore, several large retrospective cohort studies have shown that new diagnostic cases of cardiac disease are increasing in non-hospitalized patients, with the risk of major adverse cardiovascular events increasing threefold after 30 days of infection.^[6]^ Moreover, the fact that these patients had no previous underlying diseases, including hepatic or renal disease, cardiovascular disease, or other metabolic diseases, suggests that advanced age may be a key factor in the rapid progression of the disease. Studies have shown that the risk of developing complications associated with neo-coronary pneumonitis significantly increases with age, ranging from 9.9% in individuals aged 18 to 48 years to 21.9% in individuals aged ≥ 70 years.^[7]^

After infection with a novel coronavirus, including SARS-CoV-2, different types of arrhythmias similar to those seen with other coronaviruses, such as SARS-CoV and MERS-CoV, can occur. The occurrence of arrhythmias in these cases may be related to pathological changes in the heart and blood vessels post-infection, such as endothelial cell detachment and inflammation of small blood vessels throughout the body. This can lead to mixed intravascular thrombosis, thromboembolism, and infarction at corresponding sites. Additionally, cardiomyocytes may undergo degeneration, necrosis, interstitial edema, congestion, and inflammatory cell infiltration,^[8]^ explaining the elevated levels of myocardial injury markers in patients after SARS-CoV-2 infection. Direct viral invasion and immune-mediated damage can lead to inflammation and edema in the cardiac conduction tissue,^[9]^ and a positive signal for coronaviruses in the cardiac conduction system was found in pathological examinations of the myocardium of patients with SARS-CoV-2 infection.^[10]^ Necropsy studies of SARS-CoV-infected mice and rabbits have shown that myocytes from samples with cardiac damage and conduction system disease contain viral RNA.^[11]^ Currently, inflammatory factors are considered the main contributors to the development of abnormal heart rhythms, and the decrease in inflammatory markers is accompanied by a certain level of alleviation of the symptoms of conduction block occurring in patients.^[12]^ Following acute infection, the persistent viral reservoir in the heart induces a chronic inflammatory response.^[13]^ Monocyte chemoattractant protein-1 and normal T cells express increased monocyte chemoattractant protein-1, and these chemokines through the uncoupling of endothelial nitric oxide synthetase and reactive oxygen species production aggravate endothelial dysfunction and lead to recessive tissue damage, followed by chronic myocardial fibrosis, myocardial injury, and underlying arrhythmia.^[14]^ Moreover, the heart is affected by COVID-19 through the pericardial S-spike protein binding to angiotensin-converting enzyme 2 (ACE2) in host cells. ACE2 is present in various organs, including the cardiovascular system, and its decreased availability due to COVID-19 reduces its cardiovascular protective effects.^[10]^ Another potential mechanism is the incorporation of the SARS-CoV-2 genome into the DNA of infected human cells, which leads to the sustained activation of the immune and inflammation-mediated coagulation cascade. The presence of autonomic dysfunction during and after infection may explain arrhythmias.^[15]^

From the above cases, it is evident that all three patients experienced malignant arrhythmic events after the COVID-19 infection. Although the specific time sequence of SARS-CoV-2 infection and the latency period of arrhythmia occurrence could not be accurately determined at that time, it can be concluded that all three patients were infected with varying degrees of syncope caused by SARS-CoV-2. Further studies need to be conducted to clarify the average latency period of arrhythmia occurrence. Initially, it was unclear whether the malignant arrhythmia observed in these patients was temporary or permanent. However, given that all three patients exhibited syncopal symptoms, the risk of potential cardiac arrest was remarkably high for acquired atrioventricular block. Consequently, the implantation of a temporary pacemaker is necessary.^[16]^ Currently, no effective medication is available to prevent the development of malignant arrhythmias after SARS-CoV-2 infection. Therefore, rapid and effective anti-inflammation and immune response suppression in the early stages of infection may reduce the burden of long-term cardiac rehabilitation. Additionally, it is crucial to assess the cardiovascular status of asymptomatic patients, even in the absence of cardiovascular events such as arrhythmia. This is supported by previous research conducted by Hossain et al, which revealed that the frequency of symptoms may decrease as the duration of infection progresses. Moreover, they reported that 21.2% of the population experienced cardiac impairment 4 weeks after COVID-19 diagnosis, and this percentage decreased to 16.5% by 12 weeks.^[17]^ It is worth noting that the impact of COVID-19 can be long term. Serial electrocardiograms are required to monitor patients with SARS-CoV-2 infection, as arrhythmias and myocardial damage in these patients do not parallel the pulmonary abnormalities observed after a negative nasopharyngeal swab.^[18]^

The shortcomings of this case series include the following: we did not perform a cardiac biopsy on the patient, which means that we could not determine the myocardial tissue lesions in the patient. Additionally, previous autopsies of critically ill patients did not provide sufficient pathological tissue, making it difficult to identify the specific target or cause of the strain responsible for arrhythmias in the current variant of COVID-19. The local COVID-19 virus strain during this period was thought to be XBB. 1.16 Omicron subvariant, the arrhythmogenic effect of this strain still requires further basic research.

## 4. Conclusion

Appearance of fatal chronic arrhythmias following COVID-19 infection is rare, early ECG monitoring is necessary during the recovery period of COVID-19 because of the high incidence of various arrhythmias. Regular ECG review can be helpful in the early detection of evidence of malignant arrhythmia, allowing for early intervention and reduction in treatment costs. Additionally, elderly patients are particularly vulnerable to fatal arrhythmias and other cardiovascular accidents owing to their low resistance to COVID-19 and insufficient heart reserve capacity. Therefore, routine ECG monitoring and ambulatory electrocardiogram examinations should be performed as soon as possible after admission. It is important to stimulate interest in identifying the cause of arrhythmia through this case series, as the novel coronavirus is expected to coexist with human society for a long time. Finally, conducting screening of high-risk individuals, administering COVID-19 vaccinations, and performing electrocardiography for at least 8 to 12 weeks after infection, along with transthoracic echocardiography, will greatly improve clinical diagnosis and treatment.

## Author contributions

**Supervision:** Qiaowen Li.

**Writing – original draft:** Hongyun Shu.

**Writing – review & editing:** Xiaoyong Zhang, Yaqian Cui, Guojun Zhao, Xiyan Zhu.
